# The relationship between falling and fear of falling among community-dwelling elderly

**DOI:** 10.1097/MD.0000000000026492

**Published:** 2021-07-02

**Authors:** Wei-Cheng Chen, Yang-Tzu Li, Tao-Hsin Tung, Chieh Chen, Ching-Yao Tsai

**Affiliations:** a Taiwan Stipendiary Co., Ltd., Kaohsiung; b Institute of Health Policy and Management, National Taiwan University; c Department of Long Term Care, National Taipei University of Nursing and Health Science; d Taiwan Association of Health Industry Management and Development, Taipei, Taiwan; e Institute of Medical Sciences, Tzu Chi University, Hualien; f Department of Ophthalmology, Taipei City Hospital; g Institute of Public Health, National Yang-Ming University; h MS Program in Transdisciplinary Long Term Care and Bachelor's Program in Business Management, Fu Jen Catholic University, New Taipei City, Taiwan.

**Keywords:** associated factor, elderly, falling, fear of falling

## Abstract

Researchers have repeatedly examined the relationship between a previous experience of a fall and subsequent fear of falling (FOF); however, few studies have investigated the effects of falling along various timelines among older adults. The objective of this study was to determine whether experiencing a fall in the previous month or the previous year led to FOF among the elderly.

The National Health and Aging Trends Study (NHATS) in the U.S. collected information indicative of basic trends in the behavior of individuals aged 65 years and older. In the current study, we applied multiple logistic regression analysis of results from round 7 of the NHATS with the aim of identifying the risk factors associated with FOF among 5559 participants aged 65 years or older.

FOF was reported by 48.8% of those who experienced a fall in the previous year and 46.8% experienced a fall in the previous month. The results of regression analysis revealed that after adjusting for sex, age, related chronic disease, activities of daily living, and instrumental activities of daily living, FOF was significantly associated with experiencing a fall during the previous month (OR = 2.29, 95% CI: 1.78–2.95) or during the previous year (OR = 2.60, 95% CI: 2.16–3.14).

Our results indicate that experiences of falling during the previous month or the previous year were both significantly associated with a fear of falling, and caregivers should keep this in mind when dealing with community-living elderly individuals.

**Key summary points**

- Aim: The aim of this study was to determine whether the experience of falling in various timelines can lead to a fear of falling.
- Findings: Experiencing a fall during the previous month or the previous year were both significantly associated with a fear of falling, after adjusting for gender, age, related chronic disease, activities of daily living, and instrumental activities of daily living.
- Message: Care-givers should keep in mind that the elderly harbor a fear of falling for at least one year after experiencing a falling episode.

## 1 Introduction

Simple falling can have serious negative consequences for the elderly, resulting in impaired mobility, loss of independence, decreased quality of life, and even death. Previous research has indicated that every year in the United Stated, at least 12% of community-living elderly adults experience a fall of some degree, resulting in 9.9 million injuries with estimated health care costs of nearly 50 billion USD annually.^[1,2]^

It is not surprising that falling-related injuries tend to be more severe among the elderly than among younger individuals.^[3]^ Researchers have determined that the risk factors of falling can be intrinsic (related to the individual) or extrinsic (related to the environment). Intrinsic factors include age, gender, race, physical and psychological functionality, and the presence of chronic conditions. Extrinsic risk factors include hazards around the home.^[4,5]^ Recent evidenced-based studies have revealed that a fear of falling (FOF) may develop in the elderly who have experienced an accidental fall, which has been recognized as a specific health-related issue.^[6,7]^

From a clinical perspective, FOF is defined as a sense of concern regarding the dangers of falling that is sufficient to impede one's participation in daily activities.^[7]^ Moreover, a published study also demonstrated that older adults who reported lower levels of FOF were more likely to perceive quality of life (QoL).^[8]^ The objective of the current study was to determine whether one's experience of falling during the previous month or the previous year is significantly associated with a fear of falling. Therefore, experts were able to identify and implement interventions to reduce the sense of FOF among the elderly.

## 2 Methods

### 2.1 Study design and sample

The study cohort was assembled from the National Health and Aging Trend Study (NHATS) (www.nhats.org), which is a national program in the U.S. tasked with collecting information on adults aged 65 or older who are enrolled in Medicare.^[9]^ The collected information is meant to guide efforts to reduce disability, enhance independent functioning, promote health, and improve the quality of life of the elderly in America.^[9]^ NHATS is sponsored by the Division of Behavioral and Social Research, a division of the National Institute on Aging. NHATS has been collecting data annually since 2011 (Round 1). The data used in this study were collected in 2017 (Round 7), which included 6321 participants. A total of 5559 individuals were included in this investigation for FOF.

### 2.2 Measurements

The incidence of FOF was determined using a dichotomous yes/no question: *In the last month/year, did you worry about falling down?* NHATS defines falling as any incident of falling, slipping, or tripping that involves losing one's balance and landing at a lower level (e.g., on the floor or ground).

Potential risk factors for falling were categorized under the following headings: demographic characteristics, physical medical conditions, symptoms of depression, memory complaints, and physical functional capacity.^[5]^

### 2.3 Social demographic characteristics

Demographic characteristics included age and sex. Age was divided into 5 5-year groups (65–69, 70–74, 75–79, 80–84, and ≥85 years). Gender was a dichotomous variable (male/female).

### 2.4 Medical conditions

Medical conditions included previous heart attack, heart disease, high blood pressure, arthritis, osteoporosis, diabetes mellitus, lung disease, stroke, dementia, and cancer.

### 2.5 Symptoms of depression

Symptoms of depression were revealed by asking the following question: *Over the last month, how often have you felt down, depressed, or hopeless?* The answer provided 4 options: not at all, several days, more than half the days, nearly every day. The first 2 responses (not at all and several days) indicated “no symptoms of depression,” whereas the other 2 responses (more than half the days and nearly every day) indicated “symptoms of depression”

### 2.6 Memory complaints

Participants were asked to report their memory status using the following question: *First, how would you rate your memory at the present time? Would you say it is excellent, very good, good, fair, or poor?* The first 2 responses (excellent and very good) indicated “no memory complaints), whereas the other 2 responses (fair or poor) indicated the “presence of memory issues” presence of memory issues.

### 2.7 Physical functional capacity

Physical functional capacity was evaluated in terms of activities of daily living (ADL) and instrumental activities of daily living (IADL). ADL functions were used to assess the ability to perform fundamental self-care tasks. One well-known assessment for ADL developed by Katz et al in the 1960 s evaluated 6 items, such as bathing, dressing, toileting, transferring to and from a chair, maintaining continence, and eating.^[10,11]^ The IADL function was used to assess one's ability to perform tasks of living independently. Lawton's IADL is a widely used assessment used to measure the functional ability of older adults to perform complex tasks.^[12]^ Lawton's IADL assesses the ability to use a telephone, go shopping, prepare food, perform housekeeping, do one's laundry, use a designated form of transport, assume responsibility for taking one's own medication, and handle finances.^[12]^ Several studies have devised ADL and IADL measurements for NHATS data based on the early definition of Katz's indices of ADL and Lawton's indexes of IADL.^[13–16]^ In the current study, ADL functions included one's ability of eating, bathing, dressing themselves, using a toilet, transferring one's self into and out of bed, transferring one's self into and out of a chair, moving around under their own power, and climbing and ascending stairs. IADL functions included one's ability to launder clothes, prepare a hot meal, pay bills, go shopping, venture out of the house/apartment by themselves, using a cell phone, and handling medical prescriptions. All of the questions were dichotomous, and the total scores were proportional to the amount of help that the individual needed to complete the above-mentioned tasks. One of our objectives in this study was to assess the degree to which FOF limits one's ability to perform day-to-day activities; therefore, our focus was on physical function.

### 2.8 Statistical analysis

The collected data were modified for differential nonresponses and inapplicability. Chi-Squared tests were used to analyze bivariate correlations. Multiple logistic regression analysis was used to identify factors related to FOF after adjusting for confounding factors. Two models were used in this study. Model 1 was based on demographic characteristics, whereas Model 2 was based on medical conditions, symptoms of depression, memory, and the capacity to perform physical functions. Statistical significance was set at *P* < .05. Statistical analysis was performed using the Statistical Package for Social Sciences version 21.0 (SPSS Inc., Chicago, IL).

## 3 Results

**Figure 1:**
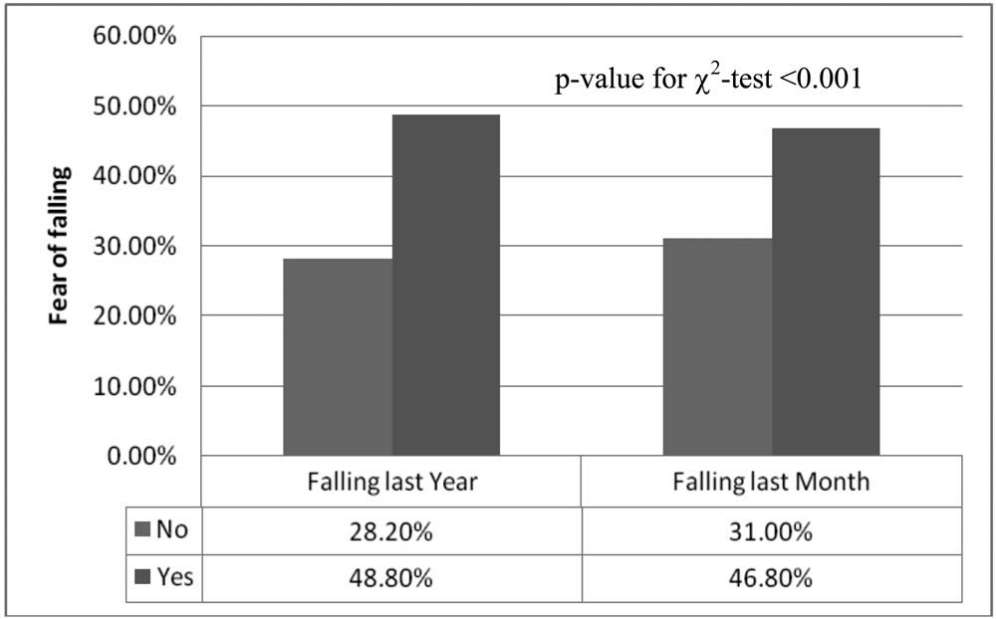
The figure represents the percentage of sample person who reported fear of falling in various timeline.

Figure 1 illustrates the relationship between previous experiences of falling and FOF. Subjects who experienced falling during the previous year were more likely to report FOF (yes: 48.8 vs no: 24.8%, *P* < .001). Subjects who experienced falling during the previous month were also more likely to report FOF (yes: 46.8% vs no: 31.0%, *P* < .001).

Table 1 lists the distributions of the baseline characteristics among the study population. Table 2 lists the results of univariate analysis. Experiencing a fall during the previous year (OR = 2.85, 95% CI: 2.49–3.25) or the previous month (OR = 1.93, 95% CI: 1.66–2.31) was significantly associated with FOF. Other significant risk factors included older age, female sex, chronic disease, higher ADL score, and higher IADL score.

**Table 1:** The distributions of baseline characteristics among study population (n = 5559).

Variables	n	%
**Social demography**		
Age		
65–69	390	7.0
70–74	1328	23.9
75–79	1248	22.5
80–84	1105	19.9
85+	1488	26.8
Gender		
female	3239	58.3
male	2320	41.7
**Health Status**		
Heart attack		
no	5400	97.3
yes	150	2.7
Heart disease		
no	4237	76.4
yes	1312	23.6
High blood pressure		
no	1480	26.6
yes	4,074	73.4
Arthritis		
no	1724	31.0
yes	3831	69.0
Osteoporosis		
no	3839	69.2
yes	1709	30.8
Diabetes mellitus		
no	3903	70.2
yes	1654	29.8
Lung disease		
no	4369	78.7
yes	1184	21.3
Stroke		
no	5398	97.2
yes	157	2.8
Dementia		
no	5056	91.0
yes	501	9.0
Cancer		
no	5184	93.4
yes	368	6.6
Symptoms of depression		
no	5122	92.7
yes	406	7.3
Memory problem		
no	3985	75.8
yes	1272	24.2
Basic disability		
ADL		
0	3005	77.9
1	723	18.7
2	28	0.7
7	38	1.0
8	51	1.3
9	13	0.3
IADL		
0	191	3.4
1	315	5.7
2	413	7.4
3	679	12.2
4	837	15.1
5	880	15.8
6	1153	20.7
7	644	11.6

ADL = activities of daily living, IADL = instrumental activities of daily living.

**Table 2:** Univariate analysis on the factors associated with fear of falling (N = 5559).

	Fear of falling		Unadjusted OR	*P* value
	no (n = 3,729)	yes (n = 1,830)	(95% CI)	
Variables	n (%)	n (%)		
Falling last y				
no	3062 (71.8)	1204 (28.2)	1.00	---
yes	658 (51.6)	618 (48.8)	2.85 (2.49–3.25)	<.001
Falling last mo				
no	3373 (69.0)	1516 (31.0)	1.00	---
yes	354 (53.2)	312 (46.8)	1.93 (1.66–2.31)	<.001
Age (y)				
65–69	305 (78.2)	85 (21.8)	1.00	---
70–74	979 (73.7)	349 (26.3)	1.28 (0.97–1.67)	.07
75–79	886 (71.0)	362 (29.0)	1.47 (1.12–1.92)	.005
80–84	715 (64.7)	390 (35.3)	1.96 (1.49–2.56)	<.001
85+	844 (56.7)	644 (43.4)	2.74 (2.10–3.55)	<.001
Gender				
female	2042 (63.0)	1197 (37.0)	1.00	---
male	1687 (72.7)	633 (27.3)	0.64 (0.57–0.71)	<.001
Heart attack				
no	3650 (67.6)	1750 (32.4)	1.00	---
yes	75 (50.0)	75 (50.0)	2.08 (1.50–2.88)	<.001
Heart disease				
no	2971 (70.1)	1266 (29.9)	1.00	---
yes	753 (57.4)	559 (42.6)	1.74 (1.53–1.97)	<.001
High blood pressure				
no	1,110 (75.0)	370 (25.0)	1.00	---
yes	2,615 (64.2)	1,459 (35.8)	1.67 (1.46–1.91)	<.001
Arthritis				
no	1340 (77.7)	384 (22.3)	1.00	---
yes	2385 (62.3)	1446 (37.7)	2.11 (1.85–2.41)	<.001
Osteoporosis				
no	2733 (71.2)	1106 (28.8)	1.00	---
yes	989 (57.9)	720 (42.1)	1.79 (1.59–2.02)	<.001
Diabetes mellitus				
no	2692 (69.0)	1211 (31.0)	1.00	---
yes	1035 (62.6)	619 (37.4)	1.32 (1.17–1.50)	<.001
Lung disease				
no	3016 (69.0)	1353 (31.0)	1.00	---
yes	710 (60.0)	474 (40.0)	1.48 (1.30–1.69)	<.001
Stroke				
no	3640 (67.4)	1758 (32.6)	1.00	---
yes	88 (56.1)	69 (43.9)	1.62 (1.17–2.23)	.002
Dementia				
No	3440 (68.0)	1616 (32.0)	1.00	---
Yes	287 (57.3)	214 (42.7)	1.58 (1.31–1.91)	<.001
Cancer				
No	3502 (67.6)	1,682 (32.4)	1.00	---
Yes	222 (60.3)	146 (39.7)	1.36 (1.10–1.70)	.003
Symptoms of depression				
No	3543 (69.2)	1579 (30.8)		---
Yes	170 (41.9)	236 (58.1)	3.11 (2.53–3.82)	<.001
Memory compliant				
No	2815 (70.6)	1170 (29.4)	1.00	---
Yes	723 (56.8)	549 (43.2)	1.83 (1.60–2.08)	<.001
ADL				
0	2379 (79.2)	626 (20.8)	1.00	---
1	413 (57.1)	310 (42.9)	2.85 (2.40–3.38)	<.001
2	15 (53.6)	13 (46.4)	3.29 (1.55–6.95)	.1
7	23 (60.5)	15 (39.5)	2.47 (1.28–4.77)	.05
8	37 (72.5)	14 (27.5)	1.43 (0.77–2.67)	.76
9	2 (15.4)	11 (84.6)	20.9 (4.62–94.54)	<.001
IADL				
0	144 (26.6)	397 (73.4)	1.00	---
1	115 (60.2)	76 (39.8)	0.80 (0.55–1.16)	.24
2	173 (54.9)	142 (45.1)	0.84 (0.59–1.20)	.35
3	419 (61.7)	260 (38.3)	1.06 (0.76–1.47)	.71
4	529 (63.2)	308 (36.8)	1.13 (0.82–1.56)	.44
5	600 (68.2)	280 (31.8)	1.41 (1.02–1.95)	.04
6	828 (71.8)	325 (28.2)	1.68 (1.22–2.31)	.001
7	480 (74.5)	164 (25.5)	1.93 (1.37–2.71)	<.001

ADL = activities of daily living, IADL = instrumental activities of daily living, OR = odds ratio.

The influence of independent risk factors on FOF was examined using a multiple logistic regression model. As shown in Table 3, subsequent to adjustment for confounding factors in Model 1, the following factors were significantly related to FOF: falling during the previous month (OR = 2.63, 95% CI: 2.21–3.13), falling during the previous year (OR = 2.75, 95% CI: 2.40–3.15), age (75–79 years vs 65–69 years, OR: 1.43, 95% CI: 1.08–1.89; 80–84 years vs 65–69 years, OR = 1.90, 95% CI: 1.44–2.51; ≧85 years vs 65–69 years, OR = 2.44, 95% CI: 1.86–3.20), and female sex (OR = 0.67, 95% CI: 0.59–0.76). After adjusting for sex, age, chronic disease, ADL score, and IADL score, falling during the previous month (OR = 2.29, 95% CI: 1.78–2.95) and falling during the previous year (OR = 2.60, 95% CI: 2.16–3.14) were both significantly associated with FOF.

**Table 3:** Multiple logistic regression model of associated factors for fear of falling.

	Fear of falling					
	Beta	SE	*P* value	OR	95% CI	
Variables					Lower	Upper
Model 1						
Falling last mo	0.98	0.09	<.001	2.63	2.21	3.13
Falling last y	1.01	0.07	<.001	2.75	2.40	3.15
Age (y)						
65–69	---	---	---	1.00	---	---
70–74	0.23	0.14	.119	1.26	0.95	1.66
75–79	0.36	0.14	.01	1.43	1.08	1.89
80–84	0.64	0.14	<0.001	1.90	1.44	2.51
85+	0.89	0.14	<.001	2.44	1.86	3.20
Male gender	–0.40	0.06	<.001	0.67	0.59	0.76
Model 2						
Falling last mo	0.83	0.13	<.001	2.29	1.78	2.95
Falling last y	0.96	0.10	<.001	2.60	2.16	3.14
Age (y)						
65–69	---	---	---	1.00	---	---
70–74	0.34	0.19	.088	1.41	0.96	2.06
75–79	0.39	0.20	.05	1.48	1.01	2.17
80–84	0.62	0.20	.002	1.85	1.26	2.73
85+	0.89	0.20	<.001	2.43	1.65	3.57
Male gender	–0.49	0.10	<.001	0.61	0.51	0.74
Heart Attack	0.50	0.27	.07	1.64	0.97	2.78
Heart Disease	0.16	0.10	.10	1.18	0.97	1.43
High Blood Pressure	0.05	0.10	.61	1.05	0.87	1.27
Arthritis	0.39	0.09	<.001	1.48	1.23	1.77
Osteoporosis	0.16	0.10	.10	1.17	0.97	1.41
Diabetes Mellitus	0.21	0.09	.02	1.23	1.03	1.47
Lung Disease	0.01	0.10	.90	1.01	0.83	1.24
Dementia	0.04	0.24	.87	1.04	0.65	1.65
Symptom of depressed	0.68	0.18	<.001	1.98	1.38	2.84
Memory Compliant	0.34	0.10	.001	1.40	1.15	1.70
ADL	0.11	0.03	<.001	1.12	1.06	1.18
IADL	–0.07	0.03	.28	0.94	0.88	0.99

Model 1: OR adjusted by age and sex. Model 2: OR adjusted by age, sex, heart attack, heart disease, high blood pressure, arthritis, osteoporosis, diabetes mellitus, lung disease, dementia, symptom of depressed, memory complaint, ADL, and IADL. ADL = activity of daily living, CI = confidence interval, IADL = instrumental activities of daily living, OR = odds ratio.

Table 4 indicates the prevalence of FOF in various populations, ranging from 11.5% to 76.6%.^[17–22]^ Four published studies listed previous experience of falling as a significant risk factor of FOF,^[17,19,20,22]^ which is in agreement with the findings of the current study. Subjects who experienced falling in the previous year or the previous month were >2 times more likely to report FOF, even after adjusting for other risk factors.

**Table 4:** Prevalence of fear of falling in various study population.

Author	Study year	Number of participants	Study age	Setting	Prevalence of fear of falling	Associate factors
Kim et al^[16]^	2013	9033	≥60 y	Korea	76.6%	previous fall, experience of body pain, self-reported health status, symptom of depressed, receipt of more doses of drugs per day, age, female gender, dependence for instrumental activities of daily living, dependence for activities of daily living, and educational level
Kumar et al^[17]^	2014	1254	≥65 y	England	19%	BMI, ethnic group, income, educational level, ability to use public transport, using walking aids, self-perceived physical health, problems with balance and inability to rise from a chair of knee height
Gazibara et al^[18]^	2017	354	≥65 y	Serbia	11.5%	Previous fall
Chang et al^[19]^	2017	3824	≥ 65 y	Taiwan	53.4%	gender, previous fall, age, insomnia, depression and worse subjective health
Liu ^[20]^	2015	445	≥ 65 y	Hong Kong	64.73%	gender, poor vision, arthritis and poor performance in various assessment tests
Pirrie et al^[21]^	2020	595	≥ 55 y	Ontario	37.4%	Previous fall, self-reported health status, alcohol consumption

## 4 Discussion

### 4.1 Clinical implications

Previous studies reported that previous experience of falling can lead to FOF;^[5,17–20,22]^ however, the actual nature of this correlation remains controversial.^[23,24]^ In the current study, we also determined that previous experience of falling was a significant risk factor for FOF, regardless of whether the fall(s) occurred in the previous month or previous year. Previous studies typically used time intervals of 6 months, 12 months, 20 months, or even 24 months;^[18–23,25]^however, at least one study used an interval of 3 months.^[26]^

Advanced age is a well-known risk factor for FOF, and our results support this assertion. Four significant issues related to physical aging include slowness, stress, homeostatic equilibrium, and pacing.^[27]^ A reduction in reserve capacity may be the main factor causing older adults to develop FOF, in which case, old age could be a predictor of FOF.

Consistent with many previous studies, we found that FOF was more common among females;^[21,28]^ however, at least one study reported contradictory findings.^[20]^ Further studies should be conducted to explore the mechanisms of gender differences in FOF development. Many studies have reported links between chronic medical conditions (e.g., poor vision and arthritis) and FOF.^[17,18,20–22,29,30]^ Depression and anxiety have also been linked to FOF inamong older adults.^[17,20,27,29,30]^ In the current study, arthritis and symptoms of depression were associated with an elevated risk of developing FOF.

It was not surprising to discover that lower activities of a daily living level are a risk factor of FOF.^[31,32]^ In this study, Model 2 identified age, gender, arthritis, symptoms of depression, ADL, and previous experience falling as significant risk factors of FOF. After adjustment for confounding factors, the present study also found that both falling in the previous month and falling the previous year were significant factors of FOF. Previous studies have identified FOF as a health issue among the elderly, as it tends to hamper one's willingness to engage in physical and/or social activities, often leading to depression and decreased quality of life.^[5,28,33–39]^ A number of medical interventions have been developed to reduce FOF among the elderly.^[40–43]^ Our results indicate that these interventions are applicable to any elder adult who experienced falling in the previous 12 months.

### 4.2 Methodological considerations

This study had a number of limitations, which should be considered in the interpretation of these findings. First, the NHATS survey does not provide data pertaining to environmental factors, footwear or clothing, walking aids or assistive devices, or gait. Second, the self-reported data from NHATS are prone to errors and recall bias, and the data were not collected for specifically deduce the link between falling and FOF. Third, this survey did not cover elderly adults residing in hospitals or nursing homes. Fourth, only one question (*in the last month, did you worry about falling down?*) was used to assess the outcomes. This could have led to misunderstandings and oversimplified the issue. Broader assessments, such as the Falls Efficacy Scale-International, are more sensitive than a single dichotomous question.^[44]^ Fifth, the ADL and IADL scales used in this study were derived from the definition of Kat's ADL and Lawton's IADL. In other words, it may limit the assessment of the ability of activities of daily living and instrumental activities of daily living among the elderly. Finally, this cross-sectional study was limited in its ability to draw causal inferences. Prospective longitudinal studies are required to overcome these limitations.

## 5 Conclusion

In conclusion, this study demonstrated that experiencing a fall during the previous month or the previous year was significantly associated with a fear of falling, after adjusting for gender, age, related chronic disease, activities of daily living, and instrumental activities of daily living. Care-givers should keep in mind that the elderly harbor a fear of falling for at least 1 year after experiencing a falling episode.

## Acknowledgment

We would like to thank the Sunflower Statistical Consulting Company, Kaohsiung, Taiwan, for the statistical advice.

## Author contributions

**Conceptualization:** Wei-Cheng Cheng, Yang-Tzu Li, Tao-Hsin Tung.

**Data curation:** Wei-Cheng Cheng, Yang-Tzu Li, Tao-Hsin Tung.

**Formal analysis:** Wei-Cheng Cheng, Tao-Hsin Tung.

**Funding acquisition:** Wei-Cheng Cheng, Tao-Hsin Tung.

**Investigation:** Wei-Cheng Cheng.

**Methodology:** Wei-Cheng Cheng, Yang-Tzu Li, Tao-Hsin Tung.

**Project administration:** Wei-Cheng Cheng, Tao-Hsin Tung.

**Resources:** Wei-Cheng Cheng.

**Software:** Wei-Cheng Cheng, Tao-Hsin Tung.

**Supervision:** Tao-Hsin Tung, Chieh Chen, Ching-Yao Tsai.

**Validation:** Wei-Cheng Cheng.

**Visualization:** Wei-Cheng Cheng.

**Writing – original draft:** Wei-Cheng Cheng.

**Writing – review & editing:** Wei-Cheng Cheng.
